# Oral Administration of L-Arginine Improves the Growth and Survival of Sow-Reared Intrauterine Growth-Restricted Piglets

**DOI:** 10.3390/ani15040550

**Published:** 2025-02-13

**Authors:** David W. Long, Barry D. Long, Gayan I. Nawaratna, Guoyao Wu

**Affiliations:** Department of Animal Science, Texas A&M University, College Station, TX 77843, USA

**Keywords:** amino acids, arginine, health, metabolism, nutrition, pigs

## Abstract

Neonatal piglets with intrauterine growth restriction (IUGR) exhibit reduced rates of growth and survival. The present study tested the hypothesis that L-arginine supplementation can mitigate this problem. One hundred and twelve (112) IUGR piglets (with a mean birth weight of 0.84 kg) from 28 sows (four IUGR piglets/sow) were assigned randomly into one of four groups. Piglets were nursed by sows and orally administered 0, 0.1, 0.2, or 0.4 g L-arginine (in the form of L-arginine-HCl) per kg body weight (BW) twice daily between 0 and 14 days of age. The total doses of L-arginine were 0, 0.2, 0.4, or 0.8 g/kg BW/day. Appropriate amounts of L-alanine were added to L-arginine solutions so that all groups of piglets received the same amount of nitrogen. Piglets were weighed on days 0, 7, and 14 of age. On day 14, blood samples (5 mL) were obtained from the jugular vein of piglets at 1 h after suckling, and their milk consumption was measured over a 10-h period using the weigh–suckle–weigh technique. Milk intake did not differ (*p* > 0.05) among the four groups of piglets. Oral administration of 0.4 g L-arginine/kg BW/day increased (*p* < 0.05) the circulating levels of arginine, creatine, and anabolic hormones (insulin, growth hormone, and insulin-like growth factor-I), but decreased (*p* < 0.05) plasma concentrations of ammonia and cortisol (a catabolic hormone). Compared to the control group, IUGR piglets administered 0.2 and 0.4 g L-arginine/kg BW/day increased (*p* < 0.05) weight gain by 19% and 31%, respectively. Growth did not differ (*p* > 0.05) between the control and 0.8 g L-arginine/kg BW/day groups. The survival rates of IUGR piglets were 50%, 75%, 89%, and 89%, respectively, for the 0, 0.2, 0.4, and 0.8 g L-arginine/kg BW/day groups. Collectively, these results indicate that the growth and survival of IUGR piglets can be improved through L-arginine supplementation.

## Simple Summary

Among livestock species, pigs have the highest rate of intrauterine growth restriction (IUGR) because of large litter size and restricted feeding programs. IUGR generally affects 15–20% and 30% of piglets from sows with 10–13 and 18 liveborn piglets per litter, respectively. All piglets may be IUGR neonates when part or all periods of pregnancy occur in warm seasons. Without proper nutritional intervention, most of them will die before preweaning due to physiological and metabolic disorders. Thus, IUGR piglets are culled at birth on most swine farms. This represents a major loss to the global pork industry and a concern over animal welfare. Results of this study indicate that daily oral administration of 0.4 g L-arginine (as L-arginine-HCl) per kg body weight between 0 and 14 days of age was effective in increasing the circulating levels of arginine, anabolic hormones (insulin, growth hormone, and insulin-like growth factor-I), and growth, while reducing plasma concentrations of ammonia, cortisol (a catabolic hormone), and mortality. Thus, L-arginine offers a promising solution to both promote growth and save IUGR piglets during the neonatal period, thereby improving the efficiency and profit of the global swine industry.

## 1. Introduction

Pigs exhibit severe naturally occurring intrauterine growth restriction (IUGR), which is defined as impaired growth and development of the mammalian embryo/fetus or its organs during pregnancy [1]. In practice, IUGR is often diagnosed as less than two standard deviations below the mean birth weight for breed and gestational age [2,3,4,5]. At birth, runt piglets may weigh only one half or even one third as much as their largest littermates [6,7,8]. IUGR piglets have a low birth weight (LBW), but LBW neonates include not only IUGR but also preterm neonates. Under current restricted feeding programs for gestating swine (e.g., 2 kg diet/day) to prevent excessive maternal fat accretion, fetuses do not receive a sufficient supply of amino acids (AAs) [9,10,11]. Thus, approximately 15–20% of piglets are born as IUGR neonates with a birthweight of <1.1 kg for breeds with Duroc, Hampshire, Landrace, and Yorkshire genetic backgrounds (having a live-born litter size of 10–13) as compared with the mean birth weight of 1.4 kg [12,13,14]. In regions (e.g., Denmark) where the average live-born litter size of sows is 18, IUGR affects ~30% of piglets [15,16,17]. When part or all periods of pregnancy occur in warm seasons (e.g., a hot summer with an ambient temperature of up to 40 °C in TX, USA), IUGR may affect 50–100% of newborn piglets. Without proper nutritional intervention, most (76%) of them will die before preweaning [18] due to physiological and metabolic disorders [19,20,21,22,23,24,25]. In addition, low birth weight is associated with reduced postnatal growth in piglets during pre- and postweaning periods [26,27,28,29,30]. To date, IUGR piglets are culled at birth on most swine farms because there are no effective nutritional means to prevent their preweaning death and growth retardation [31,32]. This is a major loss to the productivity of sows and the global pork industry, as well as a concern over animal welfare.

L-Arginine (Arg) is a nutritionally essential AA in young mammals, because its endogenous synthesis does not meet metabolic needs [33,34]. Human infants with low birth weights have high rates of mortality because of elevated ammonia concentrations in plasma due to an Arg deficiency that impairs the hepatic urea cycle [35,36,37,38]. Such a metabolic defect causing neonatal deaths also occurs in Arg-deficient piglets maintained on total parenteral nutrition [39]. Additionally, IUGR piglets have low Arg concentrations (<110 µM in plasma) and elevated ammonia concentrations (>100 µM in plasma) in the blood circulation due, in part, to reductions in both the mass of enterocytes [cells responsible for the formation of citrulline (the immediate precursor of Arg) from glutamine and proline] and the availability of *N*-acetylglutamate as a metabolic activator of the intestinal citrulline-synthetic pathway [40,41]. Thus, we hypothesized that Arg supplementation to these compromised piglets can improve their growth and survival. The present study was conducted to test this hypothesis.

## 2. Materials and Methods

The experimental protocol of this study was approved by the Texas A&M University Institutional Animal Care and Use Committee. All animals were maintained at the Texas A&M University Swine Center. The Animal Use Protocol number is 2014-0348.

### 2.1. Chemicals

L-Arginine-HCl and L-alanine were the products of Ajinomoto Inc. (Tokyo, Japan). Their purity was 99.91% and 99.94%, respectively, as analyzed by high-performance liquid chromatography (HPLC) [33,42]. HPLC-grade methanol and water were purchased from Fisher Scientific (Houston, TX, USA). Unless indicated otherwise, the sources of all other chemicals were the same as we described previously [43,44,45,46].

### 2.2. Animals

Piglets were the offspring of Yorkshire × Landrace sows bred to Duroc boars and born at term (114 days of gestation). During gestation, gilts (parity 1) and sows (parities 2–4) were fed daily 2 kg of a corn- and soybean meal-based diet containing 12% crude protein and 3083 kcal/kg metabolizable energy that met all nutrient requirements recommended by the National Research Council [47]. The numbers of gestating swine in parities 1, 2, 3, and 4 were 12, 7, 5, and 4, respectively. The total number of sows was 28. The average litter size of live-born piglets was 12. Litters with at least 4 live-born IUGR piglets/sow were used so that enough littermates were available for random assignment into one of four treatment groups within the same litter. The initial weights of the four IUGR piglets in each litter were not exactly the same. They were nursed by their own sows and were not fostered by other sows. At the start of this study, each sow nursed 12 piglets. Throughout the lactation period, sows had free access to drinking water and a corn–soybean meal-based diet containing 17.5% crude protein and 3320 kcal metabolizable energy that met all nutrient requirements recommended by the National Research Council [48]. Immediately after farrowing, all newborn piglets (day 0 of age) were weighed and then were nursed by their mothers. One hundred and twelve IUGR piglets (birth weight < 1.1 kg) with the birth weight of 842 ± 28 g (mean ± SEM) were selected from 28 litters and then assigned randomly into one of four groups (28 piglets/group) to receive oral administration of 0, 0.2, 0.4, or 0.8 g Arg/kg BW/day (see below). The number of male and female piglets within each treatment group was similar (Table 1).

### 2.3. Oral Administration of L-Arginine-HCl

Beginning at 0 day of age, immediately after weighing and nursing, piglets received oral administration of 0, 0.1, 0.2, or 0.4 g Arg (in the form of L-arginine-HCl) per kg BW via a syringe twice daily (8:00 AM and 5:00 PM) for 14 days. The total doses of supplemental Arg were 0, 0.2, 0.4, or 0.8 g/kg BW/day. L-Alanine was used as the isonitrogenous control [33]. Namely, piglets in the 0, 0.2, 0.4, or 0.8 g Arg/kg BW/day groups received the oral administration of 0.818, 0.614, 0.409, or 0 g L-alanine/kg BW twice daily, respectively. All the administered solution was swallowed by each piglet. The total doses of administered L-alanine were 1.636, 1.228, 0.818, or 0 g/kg BW/day, respectively. The dosages of Arg were chosen based on its provision (0.4 g Arg/kg BW/day) from the sow’s milk to 7-day-old suckling pigs [49]. Supplementing piglets with 0, 0.2, 0.4, or 0.8 g/kg BW/day represented 0, 50%, 100%, or 200% of Arg intake from the milk, respectively. Arg or L-alanine was dissolved in 10 mL of distilled and deionized water immediately before gavaging. Piglets were weighed at days 0, 7, and 14 of age.

### 2.4. Collection of Blood Samples from IUGR Piglets

On day 14 of the experiment, 8 piglets were randomly selected from each treatment group for blood sampling. At 8:00, 1.5 h after suckling, jugular vein blood samples (5 mL) were withdrawn from each animal into both an EDTA-containing tube and an EDTA-free tube. The blood samples were centrifuged immediately at 10,000× *g* for 1 min, and the supernatant fluid (plasma or serum) was stored at −80 °C until analyzed.

### 2.5. Determination of Milk Consumption by Piglets

At day 14 of age, after blood sampling, the milk consumption of piglets was determined between 10:00 (the initial isolation of piglets from sows) and 20:00 (the last measurement) using the weigh–suckle–weigh method as previously described [48]. Briefly, piglets were separated from their mothers every 2 h and then returned to the sows for 1 h nursing. Thus, sows did nurse piglets between 10:00 and 20:00. Each IUGR piglet in a litter was weighed before and after nursing to calculate its milk intake. This same procedure was performed five times. Our goal was not to determine the consumption of milk by piglets within 24 h. Rather, we aimed at assessing the effect of Arg supplementation on their milk intake.

### 2.6. Analysis of AAs, Metabolites, and Hormones

AAs, creatine, creatinine, and guanidinoacetate in plasma were analyzed using our established HPLC methods, as we described previously [50]. No kits were used for these chromatographic techniques. Ammonia, urea, and glucose in plasma were analyzed using glutamate dehydrogenase, urease, and hexokinase, respectively [43,44,45,46]. Assay kits were employed for the analyses of free fatty acids (Cat. #994-75409; Wako Chemicals, Richmond, VA, USA), triacylglycerols (Cat. #2780-250; Thermo DMA, Louisville, CO, USA), and total cholesterol (Cat. #2350-250; Thermo DMA).

Hormones in plasma or serum were determined as we described previously [44,45]. Briefly, total plasma concentrations of cortisol were determined using the Coat-A-Count cortisol kit (Cat. #TKCO-1; Diagnostic Products, Los Angeles, CA, USA). Insulin and growth hormone in serum were determined, respectively, using radioimmunoassay kits for porcine insulin (Cat. #PI-12K) and growth hormone (Cat. #PGH-46HK; Linco, St. Louis, MO, USA). Insulin-like growth factor-1 (IGF-1) in serum was analyzed using an assay kit (Cat. #DSL-10-2800) from Diagnostic Systems Laboratories, Inc. (Webster, TX, USA) for pigs.

### 2.7. Statistical Analysis

Results are expressed as means ± SEM. All data were first tested for normality using the Shapiro–Wilk W Test in the JMP 15 Pro software (JMP, Cary, NC, USA), and their normal distribution was confirmed by a probability of >0.05. Within each treatment group, there were no differences (*p* > 0.05) in growth or any measured metabolites between male and female piglets based on the unpaired *t*-test. Thus, sex was not included as a variable in the statistical analysis. Growth data (BW and weigh gain) were analyzed by the two-way analysis of variance, with Arg and Day as two factors [51]. Metabolic data (obtained only at one time point) were analyzed by the one-way analysis of variance [51]. Differences among treatment means were determined using the Student–Newman–Keuls multiple comparison test. Data on the rates of piglet survival were analyzed by *X*^2^ analysis [52]. A probability value of ≤0.05 was taken to indicate statistical significance.

## 3. Results

### 3.1. Milk Consumption of IUGR Piglets

The consumption of milk by IUGR piglets measured at 14 days of age was 229 ± 13, 232 ± 10, 225 ± 14, and 227 ± 11 mL/kg BW/day, respectively, in the 0, 0.2, 0.4, and 0.8 g Arg/kg BW/day groups, with a men value of 228 mL/kg BW/day. Increasing Arg supplementation from 0 to 0.8 g Arg/kg BW/day did not affect (*p* > 0.05) milk intake by IUGR piglets. Based on the content of AAs in whole sow’s milk (e.g., 1.43 g Arg and 4.08 g lysine/L) [33], the total intake of Arg by 14-day-old IUGR piglets was 327, 332, 322, and 325 mg/kg BW/day, respectively, in the 0, 0.2, 0.4, and 0.8 g Arg/kg BW/day groups, and the total intake of lysine was 934, 947, 918, and 926 mg/kg BW/day, respectively, in the 0, 0.2, 0.4, and 0.8 g Arg/kg BW/day groups. The ratio of total Arg to total lysine (g/g) consumed by IUGR piglets wase 0.350, 0.562, 0.786, and 1.21, respectively, in the 0, 0.2, 0.4, and 0.8 g Arg/kg BW/day groups.

### 3.2. Body Weights and Weight Gain of IUGR Piglets

The effects of oral administration of Arg to IUGR piglets on their growth are summarized in Table 2. Supplementation with 0.2 g Arg/kg BW/day did not affect (*p* > 0.05) the BW of piglets at 7 days of age but increased (*p* < 0.05) BW by 16% at 14 days of age, compared with the control group. At 7 and 14 days of age, the BW of piglets receiving 0.4 g Arg/kg BW/day was 20% and 24% greater (*p* < 0.05) than that for the control group, respectively. At either 7 or 14 days of age, the BW of piglets receiving 0.8 Arg/kg BW/day did not differ (*p* > 0.05) from that for the 0 or 0.2 g Arg/kg BW/day group. At 14 days of age, the BW of piglets receiving 0.8 Arg/kg BW/day was 12% lower (*p* < 0.05) than that for the 0.4 Arg/kg BW/day group.

The BW gain of piglets between 7 and 0 days of age in the 0.2 g and 0.4 Arg/kg BW/day groups was 32% and 44% greater (*p* < 0.05), respectively, than that for the control group. The BW gain of piglets between 14 and 7 days of age was 28% greater (*p* < 0.05) in the 0.4 Arg/kg BW/day group than that for the control group but did not differ (*p* > 0.05) among the other three groups of IUGR piglets. During days 14–0, the BW gain of piglets receiving 0.2 and 0.4 g Arg/kg BW/day was 24% and 35% greater (*p* < 0.05) than that for the control group, respectively, whereas the weight gain of piglets receiving 0.8 Arg/kg BW/day was 16% lower (*p* < 0.05) from that for the 0.4 Arg/kg BW/day, but did not differ (*p* > 0.05) from that for the 0 and 0.2 Arg/kg BW/day groups. During days 7–0, 14–7, and 14–0, the weight gains of piglets receiving 0.8 Arg/kg BW/day did not differ (*p* > 0.05) from those for the 0 or 0.2 Arg/kg BW/day group.

### 3.3. Survival Rates of IUGR Piglets

During the entire experimental period, the numbers of dead piglets were 14 (i.e., 6 males and 8 females), 7 (i.e., 3 males and 4 females), 3 (i.e., 2 males and 1 female), and 3 (i.e., 2 males and 1 females), in the 0, 0.2, 0.4, and 0.8 g Arg/kg BW/day groups, respectively (Table 1). The ages when death occurred were 3.8 ± 0.7 (*n* = 14), 6.9 ± 1.2 (*n* = 7), 7.7 ± 1.8 (*n* = 3), and 8.3 ± 2.0 (*n* = 3) days (means ± SEM) in the 0, 0.2, 0.4, and 0.8 g Arg/kg BW/day groups, respectively, without significant differences among the four groups of piglets. The survival rates of IUGR piglets were 50%, 75%, 89%, and 89%, respectively, for the 0, 0.2, 0.4, and 0.8 g Arg/kg BW/day groups (Table 1). The survival rates of IUGR piglets increased (*p* < 0.05) gradually with increasing the Arg dose from 0 to 0.4 g Arg/kg BW/day groups and did not differ (*p* > 0.05) between the 0.4 and 0.8 g Arg/kg BW/day groups.

### 3.4. Concentrations of AAs in Plasma

Oral administration of 0.2, 0.4, and 0.8 g Arg/kg BW/day to IUGR piglets dose-dependently increased (*p* < 0.05) the concentrations of Arg, ornithine, and proline in plasma by 34–195%, 26–76%, and 17–53%, respectively, compared with the control group (Table 3). The concentrations of citrulline in plasma were increased (*p* < 0.05) by 13% and 20% in the 0.4 and 0.8 g Arg/kg BW/day groups, respectively, compared to the control. The concentration of citrulline in the plasma of piglets receiving oral administration of 0.2 g Arg/kg BW/day was 7–12% lower (*p* < 0.05) than that for the 0.4 or 0.8 g Arg/kg BW/day group but was not different (*p* > 0.05) from the value for the control group. Oral administration of 0.2, 0.4, and 0.8 g Arg/kg BW/day to IUGR piglets decreased (*p* < 0.05) the concentrations of glutamine in plasma by 10%, 20%, and 21%, respectively, compared with the control group. Because alanine was used as the isonitrogenous control, its concentrations in plasma was the greatest (*p* < 0.05) in the control group and declined (*p* < 0.05) gradually with increasing the dose of Arg administration from 0.2 to 0.4 g/kg BW/day. Oral administration of 0.2, 0.4, and 0.8 g Arg/kg BW/day to IUGR piglets did not affect (*p* > 0.05) the concentrations of other AAs (including glycine, histidine, lysine, and methionine) in plasma, compared to the control group (Table 3).

### 3.5. Concentrations of AA Metabolites in Plasma

Data on the concentrations of AA metabolites in the plasma of IUGR piglets are summarized in Table 4. Compared with the control group, oral administration of 0.2, 0.4, and 0.8 g Arg/kg BW/day decreased (*p* < 0.05) the concentrations of ammonia in plasma by 27%, 41%, and 42%, respectively. Increasing Arg supplementation from 0 to 0.2 and 0.4 kg BW/day decreased (*p* < 0.05) the concentrations of urea in plasma by 9% and 17%, respectively, but those of urea did not differ (*p* > 0.05) between the 0 and 0.8 g Arg/kg BW/day groups. Oral administration of 0.2, 0.4, and 0.8 g Arg/kg BW/day increased (*p* < 0.05) the concentrations of creatine in plasma by 12%, 26%, and 40%, respectively, and those of guanidinoacetate by 15%, 27%, and 45%, respectively. Concentrations of glucose, creatinine, free fatty acids, triacylglycerols, or total cholesterol did not differ (*p* > 0.05) among the four groups of IUGR piglets.

### 3.6. Concentrations of Hormones in Plasma or Serum

Data on the effects of Arg supplementation on the circulating levels of hormones are summarized in Table 5. Total plasma concentrations of cortisol were reduced by 19–25% (*p* < 0.05), but serum concentrations of insulin, growth hormone, and IGF-I were increased (*p* < 0.05) by 22–29%, 24–31%, and 26–31% in IUGR piglets receiving the oral administration of 0.4–0.8 g Arg/kg BW/day, respectively, compared with the control group. Concentrations of the measured hormones did not differ (*p* > 0.05) either between the 0 and 0.2 g Arg/kg BW/day groups or between the 0.4 and 0.8 g Arg/kg BW/day groups.

## 4. Discussion

As a nutritionally essential AA for young pigs, Arg must be provided adequately in diets (e.g., milk for nursing neonates) for their optimal growth and health [53,54,55,56,57]. However, the milk of most mammals including swine is severely deficient in Arg [58,59,60,61], providing at most 40% of the Arg needed by sow-reared piglets [49]. A primary concern over raising IUGR piglets is the high rate of their mortality during the first two weeks after birth due, in part, to hypoargininemia-induced hyperammonemia [62,63,64]. Major findings of the current study are that (a) supplementing Arg at 0.2–0.8 g/kg BW/day to sow-reared IUGR piglets reduced the circulating levels of ammonia (a toxic substance at elevated concentrations) and mortality rates; and (b) supplementing Arg at 0.2–0.4 g/kg BW/day to sow-reared IUGR piglets dose-dependently enhanced their growth. Thus, Arg can provide a promising solution to successfully manage IUGR neonates.

### 4.1. Role of Exogenous Provision of Arg in Elevating the Availability of Arg and Related Metabolites in the Plasma of IUGR Piglets

Arg, along with lysine and histidine, is taken up by mammalian cells via specific transmembrane transports, including the Na^+^-independent system y^+^ (CAT-1 and CAT3) and b^0,+^AT/rBAT, as well as Na^+^-dependent transporters (e.g., ATB^0,+^) [65,66]. In neonatal mammals, including piglets, dietary Arg undergoes limited intestinal catabolism due to the near absence of arginase in enterocytes [67,68]. Because only 7–10% of Arg in blood is extracted by the liver [68,69] due to absence of the y^+^ transport system in hepatocytes [70,71], most of the diet-derived Arg is available for utilization by extrahepatic tissues in young pigs. Thus, oral administration of 0.2–0.8 g Arg/kg BW/day dose-dependently increased the plasma concentrations of Arg and its metabolites such as ornithine, proline, guanidinoacetate, and creatine in IUGR pigs (Table 3 and Table 4), as reported for pigs with normal birth weights (NBWs) [33,44]. The rate of turnover of Arg in young pigs is high, with a half-life being 0.65 h for Arg in blood [72]. The catabolism of Arg via arginase generates urea and ornithine [54]. The latter is converted into proline via ornithine aminotransferase and pyrroline-5-carboxylate reductase [54]. Additionally, Arg is a substrate for the synthesis of creatine via arginine/glycine amidinotransferase (mainly in the kidneys and pancreas) and guanidinoacetate transferase (predominantly in skeletal muscle and the liver) [50,54,73,74]. The endogenous formation of creatine compensates for its insufficient provision from sow’s milk and plays a critical role in the rapid growth and normal function of tissues, particularly skeletal muscle and brain [73,74].

In contrast to our findings, Getty et al. [75] reported that the concentrations of Arg or creatine in plasma did not differ between IUGR pigs receiving oral administration of 0 and 0.29 g Arg-HCl/kg BW/day for 16 days. Of note, these authors did not indicate the source or purity of the Arg-HCl and L-alanine used in their study nor determined the concentrations of ornithine or proline in piglet plasma. It is possible that the piglets in the previous study [75] were stressed with elevated concentrations of cortisol to induce the expression of intestinal arginase (an enzyme that degrades dietary Arg) [44]. This would result in a failure of exogenous Arg administration to increase the availability of Arg in extraintestinal tissues or the plasma concentrations of creatine and creatinine in piglets. In addition, unlike our finding that Arg supplementation did not affect the circulating levels of any branched-chain AAs in IUGR pigs (Table 3), Getty et al. [75] found that the oral administration of Arg increased the plasma concentration of valine in IUGR piglets, which is an indicator of increased muscle proteolysis because of a cortisol surge to promote the release of valine into the blood [44,76].

### 4.2. Role of Exogenous Provision of Arg in Enhancing the Growth of IUGR Piglets

Arg is not only a substrate for protein synthesis but also an activator of the mechanistic target of rapamycin (MTOR) cell signaling pathway (a master regulator of initiation of polypeptide formation) in pig cells and tissues including enterocytes, mammary epithelial cells, and skeletal muscle [77,78,79]. Thus, oral administration of Arg 0.2–0.4 g Arg/kg BW/day dose-dependently enhanced the weight gain of IUGR piglets, when compared with the control group (Table 2). To our knowledge, this is the first report for the use of Arg to improve the growth of sow-reared IUGR piglets. Of particular note, similar results have been reported for early-weaned NBW or IUGR pigs fed milk protein-based diets supplemented with 0.2%, 0.4%, 0.6%, or 1% Arg [33,78,80,81]. In contrast, Getty et al. [75] found that oral administration of Arg-HCl daily (0.29 g/kg BW/day) between 1 and 16 days of age reduced the BW of IUGR (0.69–0.92 kg of birth weights) and NBW (1.3–1.5 kg of birth weights) piglets by 22% and 13%, respectively, compared with the control piglets receiving oral administration of the isonitrogenous amount of alanine. Because Getty et al. [75] did not determine the amount of Arg or lysine in sow’s milk and its consumption by IUGR piglets, it is unknown whether the studied animals experienced nutritional antagonisms among basic AAs or inadequate nutrient intake. In addition, it is possible that the negative effect of Arg administration on IUGR piglets [75] might have resulted from the occurrence of stress, as noted above. Furthermore, in the work of Getty et al. [75], L-arginine-HCl and L-alanine solutions were prepared once per week by dissolving each AA in sterile PBS and were stored at 4 °C for daily administration into piglets without any filtration before use. In such cases, the possibility of microbial contamination in the administered AA solutions cannot be ruled out.

Oral administration of 0.8 g Arg/kg BW/day had no statistically significant effect on the weight gain of IUGR pigs in comparison with the control group (Table 2). Similarly, dietary supplementation with 1.5% Arg did not affect growth rate in IUGR pigs fed milk protein-based diets [80]. Thus, not all doses of supplemental Arg can improve piglet growth. Based on the concentrations of AAs (including lysine and histidine) and metabolites in plasma (Table 3 and Table 4), there was no evidence for AA antagonisms or imbalances in our IUGR piglets. Young pigs can tolerate a dietary Arg/lysine ratio of up to 2.5:1 [49], which is greater than the Arg/lysine ratios of 0.562 and 1.21 for IUGR pigs supplemented with 0.2 and 0.8 g Arg/kg BW/da, respectively. Although physiological concentrations of nitric oxide (NO, a metabolite of Arg) are beneficial for maintaining adequate blood flow (and thus nutrient supply) to tissues [18], an excessive production of NO (an oxidant) can inhibit protein synthesis and increase protein degradation in mammalian cells [82,83,84]. This may explain why the rate of growth was lower in piglets that received oral administration of 0.8 g/kg BW/day, in comparison with the 0.4 g/kg BW/day group (Table 2). The milk intake of IUGR piglets as measured on day 14 of age did not differ among the treatment groups. Thus, the increase in weight gain in Arg-supplemented piglets would not have resulted from a difference in milk consumption. A relatively small sample size of this study might not have provided sufficient statistical power to detect a significant difference in the response of IUGR piglets between the 0.8 g/kg BW/day group and the control group. Future research involving more IUGR piglets is warranted to verify our findings.

### 4.3. Role of Exogenous Provision of Arg in Reducing Plasma Ammonia Concentrations and Enhancing Survival in IUGR Piglets

Despite the major problems associated with IUGR piglets, the underlying mechanisms responsible for their high rates of neonatal mortality remain largely unknown. Due to the lack of this knowledge, the current management of IUGR piglets is empirical only and primarily aimed at cross-fostering among sows [5,26,85,86]. Although this technique is practiced on some swine farms, its effectiveness in increasing growth and survival of LBW piglets may be limited due to aggressive fighting between adopted and resident piglets [87,88,89,90,91].

**Figure 1:**
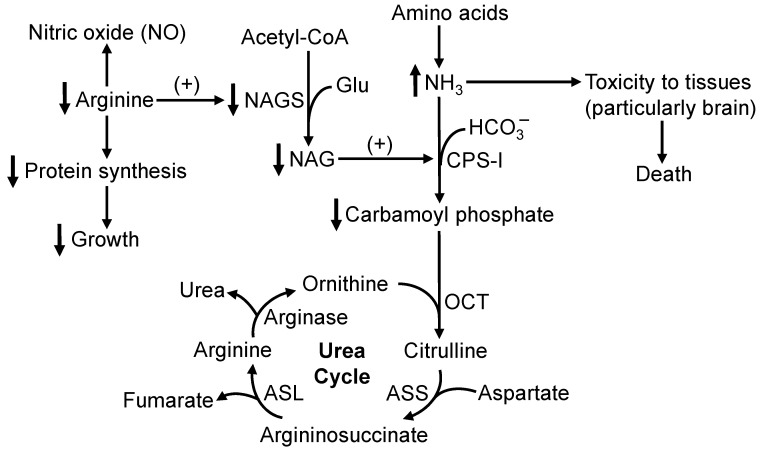
Biochemical mechanisms responsible for arginine deficiency to impair ammonia detoxification and reduce growth in IUGR piglets. Arginine is an allosteric activator of *N*-acetylglutamate synthase (NAGS), which forms *N*-acetylglutamate (NAG) from acetyl-CoA and glutamate (Glu). NAG is an allosteric activator of carbamoylphosphate synthetase-I (CPS-I), which converts ammonia) and bicarbonate into carbamoyl phosphate. Thus, when arginine is deficient, the generation of carbamoyl phosphate is decreased, thereby reducing the detoxification of ammonia as urea via the hepatic urea cycle. Elevated concentrations of ammonia are highly toxic to tissues (particularly the brain), leading to death in neonates. Additionally, as a major building block of protein and a master activator of the mechanistic target of rapamycin, the reduced availability of arginine impairs tissue protein synthesis and thus growth in animals. ↑, Increase; ↓, Decrease.

IUGR pigs do not appear to have an underdevelopment of enzymes in the hepatic urea cycle [18,40,41]. As a precursor of ornithine and an allosteric activator of *N*-acetylglutamate synthase [92], Arg is essential for the detoxification of ammonia via the urea cycle in the liver (Figure 1). Studies involving preterm and IUGR human infants have shown that hyperammonemia due to hypoargininemia and subsequent defects in the hepatic urea cycle is often a major cause of death during the neonatal period [35,36,37,38]. Elevated concentrations of ammonia in blood promote the conversion of α-ketoglutarate into glutamine in tissues (e.g., liver and skeletal muscle) via glutamate dehydrogenase and glutamine synthetase [93,94,95], therefore increasing the circulating levels of glutamine [96,97,98]. This would deplete the intermediates of the Krebs cycle for the oxidation of acetyl-CoA to provide energy in tissues [95], while reducing the production of NO (the major vasodilator) by endothelial cells [99,100] as well as blood flow and O_2_ supply to the brain [96,97], leading to hypoxia and damage in tissues. Under these clinical conditions, Arg administration prevents hyperammonemia and deaths in these compromised neonates [35,36,37,38]. Likewise, this simple method was highly effective in enhancing the activity of the hepatic urea cycle for ammonia removal, thereby decreasing the circulating levels of ammonia and enhancing the rate of survival in sow-reared IUGR piglets (Section 3). To our knowledge, this is the first report for the use of Arg to save the lives of livestock neonates. In IUGR piglets, increasing the production of ammonia from the oxidation of AAs by augmenting dietary protein intake increased the rate of their mortality [63], and such a nutritional practice should not be adopted for their feeding. Thus, activating the hepatic urea cycle through Arg administration rather than increasing the intake of all AAs or protein can be a promising means for the successful management of IUGR neonates.

Urea is a nontoxic substance in animals. As reported for NBW pigs [33], Arg supplementation to IUGR piglets reduced the concentration of urea in plasma (Table 4). This is because Arg increases the use of AAs for tissue protein synthesis and growth, therefore decreasing the availability of AAs for catabolism, ammonia generation, and thus urea production. As a result of reduced ammonia concentration in plasma, the formation of glutamine and its plasma concentrations (remaining within physiological ranges) [101] were decreased in Arg-supplemented IUGR piglets (Table 4). In laboratories and swine units, ammonia and urea in plasma or serum can be readily measured through standard procedures [102]. Thus, circulating levels of ammonia may be a useful biomarker for hepatic function and AA nutritional state in IUGR neonates.

A reduction in preweaning death loss can increase the number of pigs weaned, sow’s productivity, reproductive efficiency, and revenue. This occurs without affecting the costs associated with the feeding or management of the sow herd. Estienne and Niblett [103] have recently estimated that, on a 50-sow farm, saving one extra piglet/litter can bring about an extra revenue of USD 3000/year (assuming 2 litters/sow/year and a price of USD 30/weaned piglet). The benefit would be USD 3600/sow/year for saving one extra neonatal piglet based on 2.4 litters/sow/year.

### 4.4. Role of Exogenous Provision of Arg in Modulating the Secretion of Hormones in IUGR Piglets

Acute intravenous administration of Arg (e.g., >2 g/kg BW over 15–30 min) can stimulate the release of insulin from pancreatic β-cells via the action of NO [104] and increase the circulating levels of this hormone in mammals, including humans, ruminants, and pigs [105,106,107]. Similar results have been reported for Arg in promoting the secretion of growth hormone from the anterior pituitary gland [107]. Likewise, dietary supplementation with appropriate doses of Arg increased the circulating levels of insulin and growth hormone in NBW [33,81] and IUGR [78,80] piglets, as well as the circulating levels of IGF-I (released by the liver) in sow-reared IUGR piglets (Table 5). Interestingly, no such effects were observed in 4-month-old pigs receiving dietary supplementation with 2% Arg between 30 and 121 days of age [45]. Thus, neonatal pigs are more sensitive to the Arg stimulation of secretion of anabolic hormones than older pigs. Increases in the circulating levels of insulin, growth hormone, and IGF-I can mediate, in part, the effect of Arg administration in promoting piglet growth. However, increases in these hormones alone are not sufficient to bring about an anabolic response based on our finding that the weight gain of IUGR pigs did not differ between the 0.2 and 0.8 g Arg/kg BW/day groups.

Cortisol is the major glucocorticoid in pigs and is released in response to external and internal factors acting on the hypothalamus and pituitary glands [76]. An increase in its circulating level is a useful indicator of stress in animals [76]. As reported for early-weaned NBW piglets [81], dietary Arg supplementation reduced the plasma concentration of cortisol [released from the adrenal cortex (the outer layer of the adrenal gland)] in sow-reared IUGR piglets (Table 5). There are suggestions that NO or Arg itself (a) attenuates the release, from the hypothalamus, of corticotropin-releasing hormone, which is responsible for the release of adrenocorticotropic hormone (ACTH) from the anterior pituitary gland; and/or (b) interferes with the action of ACTH on the adrenal cortex via specific receptors (e.g., type 2 melanocortin receptors) [81,108,109]. A physiological surge of cortisol in piglets at birth and weaning is not associated with an increase in the circulating levels of glucose [1,44]. Regulation of glucose homeostasis depends on not only exogenous glucose provision and endogenous synthesis but also whole-body glucose utilization that involves complex interactions among hormones (e.g., insulin and cortisol) and signaling molecules (e.g., NO and homoarginine) [46,110]. Future studies are warranted to elucidate the underlying mechanisms. Nonetheless, a reduction in cortisol can attenuate muscle proteolysis and whole-body AA oxidation, favoring protein accretion and lean tissue growth in IUGR piglets [111,112], as reported for older pigs [113,114] and other mammals [115,116].

## 5. Conclusions

Results of this study indicate that oral administration of 0.2 or 0.4 g Arg/kg BW/day was effective in enhancing the weight gain of sow-reared IUGR piglets. Supplementation with 0.2, 0.4, or 0.8 g Arg/kg BW/day to these compromised neonates reduced their plasma ammonia concentrations and mortality rates, and supplementation with 0.4 or 0.8 g Arg/kg BW/day reduced plasma concentrations of cortisol, while increasing the circulating levels of insulin, growth hormone, and IGF-Il in these piglets. Oral administration of Arg did not affect the concentrations of free fatty acids, triacylglycerols, or total cholesterol in plasma. Thus, Arg offers a promising solution to both promote growth and save IUGR piglets during the neonatal period, thereby improving the efficiency and profits of global swine industry.

## Figures and Tables

**Table 1:** Numbers of IUGR piglets in treatment groups at the beginning and end of the experiment.

L-Arginine-HCl Supplementation (g Arg/kg BW/day)	Body Weight at Birth ^1^ (g)	Number of Piglets at the Beginning of the Experiment			Number of Piglets at the End of the Experiment			Survival Rate During the Entire Period (%)
		Total	Male	Female	Total	Male	Female	
0	838 ± 27	28	14	14	14	8	6	50
0.2	835 ± 25	28	13	15	21	10	11	75 ^†^
0.4	844 ± 21	28	14	14	25	12	13	89 ^†^
0.8	842 ± 28	28	15	13	25	13	12	89 ^†^

^1^ Data for birth weight are means with SEM, *n* = 28. The numbers of piglets in each sex at the beginning and end of the experiment are given. Piglets with intrauterine growth restriction (IUGR) received oral administration of 0, 0.2, 0.4, or 0.8 g L-arginine (Arg; as L-arginine-HCl)/kg body weight (BW) per day between 0 and 14 days of age. L-Alanine was used as the isonitrogenous control. ^†^ Different from the control group (*p* < 0.05).

**Table 2:** Effects of oral administration of L-arginine-HCl on the growth of sow-reared IUGR piglets ^1^.

L-Arginine-HCl Supplementation (g Arg/kg BW/day)	Number of Pigs (*n*)	Body Weight (kg)			Daily Weight Gain (g/day)		
		Day 0	Day 7	Day 14	Days 7–0	Days 14–7	Days 14–0
0	14	0.86 ± 0.04	1.62 ± 0.10 ^b^	2.79 ± 0.12 ^c^	109 ± 9.8 ^c^	167 ± 6.2 ^b^	138 ± 7.1 ^c^
0.2	21	0.86 ± 0.03	1.87 ± 0.06 ^a,b^	3.25 ± 0.13 ^a,b^	144 ± 7.9 ^a,b^	197 ± 12 ^a,b^	171 ± 8.6 ^a,b^
0.4	25	0.85 ± 0.02	1.95 ± 0.07 ^a^	3.45 ± 0.10 ^a^	157 ± 7.6 ^a^	214 ± 7.4 ^a^	186 ± 5.9 ^a^
0.8	25	0.86 ± 0.03	1.77 ± 0.06 ^a,b^	3.04 ± 0.09 ^b,c^	129 ± 8.0 ^b,c^	182 ± 11 ^a,b^	156 ± 6.5 ^b,c^

^1^ Data are means with SEM, *n* = 14 to 25 per group as indicated. Piglets with intrauterine growth restriction (IUGR) received oral administration of 0 (control), 0.2, 0.4, or 0.8 g L-arginine (Arg; as L-arginine-HCl)/kg body weight (BW) per day between 0 and 14 days of age. L-Alanine was used as the isonitrogenous control. Piglets were freely nursed by sows. ^a–c^: Within a column, means not sharing the same superscript letter differ (*p* < 0.05). Body weights of piglets increased (*p* < 0.05) between days 7 and 14. There was no significant interaction (*p* > 0.05) between treatment and age for either variable.

**Table 3:** Concentrations of amino acids in the plasma of sow-reared IUGR piglets receiving oral administration of L-arginine-HCl ^1^.

Amino Acid Concentration in Plasma (µmol/L)	Oral Administration of L-Arginine-HCl (g L-Arginine/kg BW/day)			
	0	0.2	0.4	0.8
Aspartate	15 ± 0.7	16 ± 0.6	15 ± 0.7	16 ± 0.8
Glutamate	154 ± 8.4	150 ± 7.0	152 ± 6.5	156 ± 7.7
Asparagine	92 ± 4.2	90 ± 3.3	89 ± 2.5	90 ± 3.8
Serine	241 ± 7.8	237 ± 8.6	244 ± 9.2	235 ± 7.3
Glutamine	683 ± 22 ^a^	615 ± 18 ^b^	547 ± 13 ^c^	539 ± 10 ^c^
Histidine	91 ± 4.0	89 ± 3.6	92 ± 4.2	91 ± 4.4
Glycine	581 ± 7.6	575 ± 8.6	571 ± 11	567 ± 9.5
Threonine	215 ± 8.3	211 ± 11	209 ± 9.7	206 ± 8.6
Citrulline	54 ± 1.2 ^d^	57 ± 1.3 ^c,d^	61 ± 1.0 ^b^	65 ± 1.4 ^a^
Arginine	86 ± 3.7 ^d^	115 ± 5.3 ^c^	141 ± 5.9 ^b^	167 ± 7.3 ^a^
β-Alanine	7.1 ± 0.4	7.0 ± 0.5	6.7 ± 0.3	6.8 ± 0.4
Taurine	129 ± 5.8	126 ± 6.5	124 ± 7.1	131 ± 7.5
Alanine	953 ± 30 ^a^	815 ± 19 ^b^	697 ± 15 ^c^	673 ± 12 ^c^
Tyrosine	158 ± 6.1	155 ± 4.7	152 ± 6.8	156 ± 5.3
Tryptophan	43 ± 1.4	42 ± 1.0	41 ± 1.6	42 ± 1.3
Methionine	71 ± 2.9	73 ± 2.1	72 ± 2.6	73 ± 3.2
Valine	229 ± 10	226 ± 8.2	223 ± 9.4	219 ± 8.9
Phenylalanine	86 ± 4.2	87 ± 3.8	84 ± 3.4	88 ± 3.1
Isoleucine	115 ± 6.3	110 ± 4.6	113 ± 5.9	112 ± 6.6
Leucine	175 ± 7.2	173 ± 8.3	177 ± 7.0	172 ± 8.6
Ornithine	58 ± 2.3 ^d^	73 ± 2.7 ^c^	87 ± 4.1 ^b^	102 ± 5.3 ^a^
Lysine	207 ± 11	203 ± 7.6	197 ± 9.0	194 ± 9.5
Cysteine	169 ± 8.5	167 ± 5.6	165 ± 8.2	163 ± 7.2
Proline	471 ± 17 ^d^	552 ± 19 ^c^	638 ± 20 ^b^	718 ± 24 ^a^

^1^ Values are means ± SEM, *n* = 8. Piglets with intrauterine growth restriction (IUGR) received oral administration of 0 (control), 0.2, 0.4, or 0.8 g L-arginine (as L-arginine-HCl)/kg body weight (BW) per day between 0 and 14 days of age. L-Alanine was used as the isonitrogenous control. Piglets were freely nursed by sows. At 14 days of age, blood samples were obtained from the jugular vein of piglets in each treatment group. Plasma was analyzed for amino acids. ^a–d^: Within a row, means not sharing the same superscript letter differ (*p* < 0.05).

**Table 4:** Concentrations of amino acid metabolites in the plasma of sow-reared IUGR piglets receiving oral administration of L-arginine-HCl ^1^.

Metabolite Concentration in Plasma (µmol/L)	Oral Administration of L-Arginine-HCl (g L-Arginine/kg of BW/day)			
	0	0.2	0.4	0.8
Ammonia ^2^	138 ± 5.6 ^a^	101 ± 4.8 ^b^	86 ± 4.3 ^c^	84 ± 4.1 ^c^
Urea	2206 ± 63 ^a^	2014 ± 41 ^b^	1825 ± 49 ^c^	2179 ± 52 ^a^
Glucose	5613 ± 122	5538 ± 140	5521 ± 103	5648 ± 154
Creatine	240 ± 7.0 ^d^	269 ± 7.6 ^c^	302 ± 8.2 ^b^	336 ± 9.5 ^a^
Creatinine	36.6 ± 1.3	38.3 ± 1.0	39.2 ± 1.3	40.9 ± 1.6
Guanidinoacetate	42.1 ± 1.4 ^d^	48.4 ± 1.7 ^c^	53.6 ± 2.0 ^b^	61.0 ± 2.5 ^a^
Free fatty acids	266 ± 15	271 ± 20	274 ± 17	264 ± 13
Triacylglycerols	776 ± 52	805 ± 66	783 ± 59	755 ± 63
Total cholesterol	1938 ± 113	1886 ± 141	1956 ± 124	2011 ± 158

^1^ Values are means ± SEM, *n* = 8. Piglets with intrauterine growth restriction (IUGR) received oral administration of 0 (control), 0.2, 0.4 or 0.8 g L-arginine (as L-arginine-HCl)/kg body weight (BW) per day between 0 and 14 days of age. L-Alanine was used as the isonitrogenous control. Piglets were freely nursed by sows. At 14 days of age, blood samples were obtained from the jugular vein of piglets in each treatment group. Plasma was analyzed for amino acid metabolites. ^2^ NH_4_^+^ plus NH_3_. ^a–d^: Within a row, means not sharing the same superscript letter differ (*p* < 0.05).

**Table 5:** Concentrations of hormones in the plasma or serum of sow-reared IUGR piglets receiving oral administration of L-arginine-HCl ^1^.

Hormone Concentration in the Circulation	Oral Administration of L-Arginine-HCl (g L-Arginine/kg BW/day)			
	0	0.2	0.4	0.8
Cortisol in plasma, nmol/L	68.5 ± 3.7 ^a^	62.6 ± 3.1 ^a,b^	55.3 ± 2.5 ^b,c^	51.6 ± 2.9 ^c^
Insulin in serum, pmol/L	62.9 ± 2.6 ^c^	67.8 ± 3.3 ^b,c^	76.6 ± 4.1 ^a,b^	81.0 ± 4.4 ^a^
Growth hormone in serum, pmol/L	370 ± 14 ^c^	412 ± 19 ^b,c^	457 ± 25 ^a,b^	485 ± 22 ^a^
IGF-I in serum, µg/L	29.7 ± 1.0 ^c^	33.6 ± 1.4 ^b,c^	37.4 ± 1.7 ^a,b^	38.8 ± 1.5 ^a^

^1^ Values are means ± SEM, *n* = 8. Piglets with intrauterine growth restriction (IUGR) received oral administration of 0 (control), 0.2, 0.4, or 0.8 g L-arginine (as L-arginine-HCl)/kg body weight (BW) per day between 0 and 14 days of age. L-Alanine was used as the isonitrogenous control. Piglets were freely nursed by sows. At 14 days of age, blood samples were obtained from the jugular vein of piglets in each treatment group. Plasma was analyzed for cortisol, whereas serum was analyzed for insulin, growth hormone, and insulin-like growth factor-I (IGF-I). ^a–c^: Within a row, means not sharing the same superscript letter differ (*p* < 0.05).

## Data Availability

All data are contained within this article.
